# The antifungal effects and mechanical properties of silver bromide/cationic polymer nano-composite-modified Poly-methyl methacrylate-based dental resin

**DOI:** 10.1038/s41598-017-01686-4

**Published:** 2017-05-08

**Authors:** Yu Zhang, Yin-yan Chen, Li Huang, Zhi-guo Chai, Li-juan Shen, Yu-hong Xiao

**Affiliations:** 1 Department of Stomatology, Kunming General Hospital of Chengdu Military Command, Teaching Hospital of Kunming Medical University, Kunming, Yunnan China; 2 Center for Dental Research, School of dentistry, Loma Linda University, Loma Linda, California USA; 3 State Key Laboratory of Military Stomatology & National Clinical Research Centre for Oral Diseases & Shaanxi key Laboratory of Stomatology, Department of Prosthodontics, School of Stomatology, Fourth Military Medical University, Xi’an, Shaanxi China

## Abstract

Poly-methyl methacrylate (PMMA)-based dental resins with strong and long-lasting antifungal properties are critical for the prevention of denture stomatitis. This study evaluated the antifungal effects on *Candida albicans* ATCC90028, the cytotoxicity toward human dental pulp cells (HDPCs), and the mechanical properties of a silver bromide/cationic polymer nano-composite (AgBr/NPVP)-modified PMMA-based dental resin. AgBr/NPVP was added to the PMMA resin at 0.1, 0.2, and 0.3 wt%, and PMMA resin without AgBr/NPVP served as the control. Fungal growth was inhibited on the AgBr/NPVP-modified PMMA resin compared to the control (P < 0.05), and the antifungal activity increased as the incorporation of the AgBr/NPVP antimicrobial composite increased. Confocal laser scanning microscopy (CLSM) showed that the number of fungal cells attached to the modified PMMA resin was considerably lower than in the control. The relative growth rate of HDPCs of modified groups were higher than 75%. The flexural strength (FS) and flexural modulus (FM) were not significantly different (P > 0.05) between the experimental and control groups. These data indicate that the incorporation of AgBr/NPVP conferred strong and long-lasting antifungal effects against *Candida albicans* to the PMMA resin, and it has low toxicity toward HDPCs, and its mechanical properties were not significantly affected.

## Introduction

Full and removable partial dentures have been used widely in oral clinical applications as traditional prosthetic methods, but they cause several oral diseases, especially among the elderly. Approximately 70% of patients may suffer from denture stomatitis^1–3^, mostly associated with *Candida albicans* (*C. albicans*)^4, 5^. It has been reported that oral bacteria readily adhere to the surfaces of dentures due to their rough surfaces and hydrophobic properties. Moreover, *C. albicans* is resistant to conventional antifungal agents, which make denture stomatitis a problem in oral treatment^6^. Therefore, denture base resins with antifungal activity might be an efficient approach to reduce the prevalence of denture stomatitis^7, 8^.

Ideal antimicrobial agents should have the characteristics of a broad spectrum, high efficiency, and long-lasting antimicrobial activity, as well as stable and excellent biocompatibility. Inorganic antimicrobial agents have been used widely in oral materials because of their excellent biological safety, stability and durable antimicrobial properties. As an antimicrobial agent, silver ions have a broad antimicrobial spectrum and can inhibit the growth of fungi, Gram-positive bacteria, Gram-negative bacteria, and viruses^9^. Additionally, silver ions have low toxicity to mammalian cells^10^ and do not cause microbial resistance^11^. The antimicrobial mechanism of silver nanoparticles is that silver ions leach from the carrier materials and interact with the peptidoglycan cell wall^12^, thus causing changed structure, increased membrane permeability, and cell death^13^. Furthermore, silver nanoparticles interact with the exposed sulfhydryl groups in bacterial proteins, thus preventing DNA replication^14^. Therefore, many studies have incorporated silver nanoparticles into denture base resins to prevent denture stomatitis^15, 16^ or into dental resins to diminish biofilm accumulation over the composite and in the restoration margins^17–19^.

Quaternary ammonium salts are a general class of compounds. They have favorable antimicrobial properties compared with other antimicrobial agents but also have the advantages of strong permeability, stable performance, low toxicity, minor skin irritation, light corrosion, and long-lasting biological effects. Therefore, they have been widely used in industry, the pharmaceutical industry and other fields^20, 21^. In recent years, studies of the quaternary ammonium antimicrobial monomer used in methyl methacrylate-based resin systems have increased. Researchers have hoped to obtain durable antimicrobial properties by incorporating quaternary ammonium salts into a methyl methacrylate-based resin system to prevent denture stomatitis^22, 23^.

Many studies have reported that denture base resins have antimicrobial activity when silver ions or quaternary ammonium antimicrobial monomers were added individually^24–27^. The approach of incorporating composites of silver ions combined with antimicrobial quaternary ammonium monomers into denture base resins has not been previously reported. In the present study, we used *C. albicans*, which is the main pathogen of denture stomatitis, as a representative strain, and then evaluated the antimicrobial activity of unpolymerized AgBr/NPVP and modified room temperature-cured denture base resins against *C. albicans* with a newly developed organic-inorganic composite antimicrobial agent, quaternary ammonium grafted AgBr nano-composite (AgBr/NPVP). In addition, the mechanical properties of the modified resins were examined.

## Materials and Methods

### Preparation and characterization of AgBr/NPVP nano-composite

The raw material for the synthesis process, including poly (4-vinylpyridinium) (PVP, Mw ~30,000), silver p-toluenesulfonate (AgPTS), nitromethane, 1-bromohexane, diethyl ether, and dimethyl sulfoxide (DMSO), were purchased from Shanghai Aladdin Chemistry Co. Ltd. (China), except for the PVP, which was provided by Nanjing University of Science and Technology. All reagents were used without further purification. The AgBr/NPVP was synthesized according to the method of ref. 28 and was characterized by X-ray diffraction (XRD, XRD-7000, Shimadzu, Japan) and transmission electron microscopy (TEM, FEI, Hillsboro, OR, USA). While PVP and NPVP were characterized by fourier transform infrared spectroscopy (FTIR, IR-prestige 21, Shimadzu, Japan), and ^1^H nuclear magnetic resonance (NMR, Bruker AVANCE III HD, Bruker-Biospin, Karlsruhe, Germany). FTIR with a resolution of 4 cm^−1^ was recorded in the 4000 cm^−1^ to 500 cm^−1^ regions, and 32 accumulative scans were collected. Five hundred megahertz of ^1^HNMR spectra were recorded on a Bruker AVANCE III NMR Fourier transform spectrometer, using deuterated DMSO as solvent. The measurement of XRD was performed in the 2θ ranges of 10–80° with a step width of 0.05°.

### Preparation of poly-methyl methacrylate (PMMA)-based dental resin specimens containing AgBr/NPVP

AgBr/NPVP and PMMA powder were mixed and homogenized in a ball mill for 8 h to produce a master batch of antifungal PMMA powder containing AgBr/NPVP at a final concentration of 1 wt%. Then, different amounts of PMMA powder were added to obtain the required concentrations of 0.1, 0.2, and 0.3 wt%. PMMA resin without AgBr/NPVP served as a control, and polyethylene (PE) film (Interscience, France) with a diameter of 10 mm was used as the negative control. The resin specimens (diameter: 10 mm, height: 1.5 mm) were prepared according to the manufacturer’s instructions and were polished with No. 600, 1000, and 1500 abrasive paper. The specimens were cleaned ultrasonically for 20 min before immersion in deionized water for 24 h at 37 °C. The aging specimens were soaked in artificial saliva at 37 °C for 1, 2, 3, or 4 w. The artificial saliva was changed every day. Before the experiment, the specimens were washed in sterile water and the surfaces were wiped with a 70% ethanol solution. After 1 min, they were washed with sterilized water and sterilized with ethylene oxide.

### Antifungal properties

#### Fungal strain and culture conditions

*C. albicans* ATCC90028 was cultured at 37 °C in Sabouraud dextrose broth (Oxoid, England) in an air incubator. Then, the overnight culture was adjusted to 0.5 McFarland and diluted to the required concentration for subsequent experiments.

#### Determination of MIC and MFC

AgBr/NPVP was dispersed in Sabouraud dextrose broth to prepare the starting fungal suspension (concentration: 10000 µg/ml). Then, serial two-fold dilutions were prepared into 50 µl volumes of Sabouraud dextrose broth in 96-well plates (Corning, New York, USA). Overnight cultures of *C. albicans* were adjusted to 2 × 10^5^ colony-forming units (CFU)/ml in Sabouraud dextrose broth^29^, and 50 µl of fungal inoculum was inoculated into each well containing AgBr/NPVP dilution broth. The same amount of broth with 50 µl of fungal suspension served as the negative control. The positive control contained an antifungal suspension, but it was a free fungal suspension. The same amount of broth was used as the blank control. The wells were read for visible turbidity after 24 h of incubation. The minimum inhibitory concentration (MIC) was defined as the endpoint where no turbidity could be detected with reference to negative, positive and blank controls. An aliquot of 10 µl from each tested well without turbidity was spread onto Sabouraud dextrose agar (SDA) plates. After 24 h of incubation, plates that contained no fungal colonies were recorded. The minimum fungicidal concentration (MFC) value was determined as the lowest concentration of the antifungal agent that produced no colonies on the plate. The tests were repeated in triplicate.

#### Fe-SEM observation

The mixtures of AgBr/NPVP suspension of 1 × MFC value with *C. albicans* ATCC90028 suspension of 2 × 10^5^ CFU/ml were extracted after 24 h. The samples were centrifuged at 8000 rpm for 3 min, fixed with 2.5% glutaraldehyde overnight at 4 °C, dehydrated with a graded ethanol series, dried in a critical-point drier, and coated with gold. The specimens were scanned by a field emission scanning electron microscope (Fe-SEM, S-4800, Hitachi, Tokyo, Japan) to observe the fungal morphologies. At the same time point, a fungal suspension without the antifungal agent AgBr/NPVP served as the control. All assays were performed in triplicate on three different occasions to ensure reproducibility.

#### Direct contact test (DCT)

The antifungal activity of the AgBr/NPVP-modified PMMA-based resin specimens was detected by DCT. All of the specimens before and after aging were embedded in sterile agar with slight pressure, and the top surface was kept higher than the surface of agar in order to prevent the specimens from moving during the experiments. Approximately 10 μl of a 1 × 10^6^ CFU/ml *C. albicans* suspension was inoculated on the surface of each specimen and then covered with PE film to prevent the fungal liquid from volatilizing and to keep it in uniform contact. The PE film served as the negative control. After 24 h of incubation at 37 °C, the specimens and covered films were transferred into sterilized tubes containing 10 ml of phosphate-buffered solution (PBS) to harvest the biofilms using sonication (3510R-MTH, Branson, Danbury, CT) for 5 min, followed by vortexing at 2400 rpm for 30 seconds using a vortex mixer (Fisher Scientific, Pittsburgh, PA). To estimate colony formation, fungal suspensions from each specimen were diluted serially, and an aliquot of 100 μl was inoculated onto SDA plates. The number of colonies was counted after 24 h. The antifungal ratio was calculated according to the following formula:

1 $${\rm{r}}( \% )=[({\rm{b}}-{\rm{c}})/{\rm{b}}]\times 100 \% $$

where r is the antifungal ratio, b is the average number of CFU recovered for the control groups, and c is the average number of CFU recovered for the groups containing AgBr/NPVP. Nine specimens were tested for each group.

#### Fungal biofilm formation and live/dead assay

All of the specimens before and after aging were placed in 24-well plates, and 1 ml of 1 × 10^6^ CFU/ml of a *C. albicans* suspension was added into each well. After incubation at 37 °C for 24 h, the specimens were transferred to fresh 24-well plates filled with fresh medium and incubated for another 24 h. Then, the fungal biofilms on the specimens were gently washed 3 times with PBS and stained using the BacLight live/dead bacterial viability kit (L13152, America). Live fungi were stained with Syto 9 to produce a green fluorescence, and fungi with compromised membranes were stained with propidium iodide to produce a red fluorescence. Live and compromised fungi that were closely associated were orange or yellow. Specimens were examined using confocal laser scanning microscopy (CLSM, FV1000, Olympus Corp, Tokyo, Japan), and 3 random fields were observed for each specimen.

### Cytotoxicity Assay

#### Cell culture

Human dental pulp cells (HDPCs) were isolated from dental pulp tissue of non-carious orthodontic teeth from young healthy patients according to a protocol verbally approved by the Ethics Committee of the Fourth Military Medical University. The cells were cultured in Dulbecco’s modified Eagle’s medium (DMEM, Gibco, USA) containing 10% fetal bovine serum, 100 IU/ml penicillin and 100 IU/ml streptomycin at 37 °C and 5% CO_2_. The medium was changed every 3 days. Cells from the third passage were used for the cytotoxicity assay.

#### Preparation of the extract

The extract of the PMMA resin was prepared by soaking each specimen in 2 ml of DMEM in the 24-well cell plate at 37 °C for 24 h. The eluent was filtered for sterilization and then stored at 4 °C before use. Five specimens were tested for each group (control, 0.1, 0.2, 0.3% AgBr/NPVP modified PMMA resin before and after aging 1, 2, 3, and 4 weeks).

#### WST-8 Assay of eluent

A WST-8 assay was used to evaluate cell viability (CCK-8, Dojindo, Kunamoto, Japan). HDPCs were inoculated into the wells of 96-well micro plates at a density of 2 × 10^3^ cells per well. After 24 h of incubation, the culture medium in the 96-well plates was removed and replaced with 100 μl of eluent of PMMA resin. The cells were cultured for another 24 h at 37 °C, the medium was aspirated, and cells were washed twice with phosphate-buffered saline (PBS). Subsequently, medium (100 μl) and CCK-8 solution (10 μl) were added to each well. After incubation for 2 h, the absorbance at 450 nm was measured with a microplate reader (Bio-Rad Laboratories, Hercules, CA, USA). Blank and medium controls were treated identically. The results are expressed as optical density (OD) values after blank (medium only) correction. The relative growth rate (RGR) of the cells was calculated according to the following equation:

2 $${\rm{RGR}}( \% )={A}_{{\rm{test}}}/{A}_{{\rm{blank}}}\times 100 \% $$

where *A* is the absorbance value read from the microplate reader. Reported values are the means of three replicates.

### Mechanical properties

#### Degree of Conversion (DC)

The degree of auto-polymerization conversion of the control and experimental PMMA resin specimens was measured by FTIR. The FTIR, whose resolution was 4 cm^−1^, was recorded in the 1800 cm^−1^ to 1550 cm^−1^ regions, and 24 accumulative scans were started after 5 min, 10 min, 20 min, 30 min, 1 h, 4 h and 24 h when the PMMA resin powder and liquid were mixed. The peak height at 1638.6 (C = C) and 1720 cm^−1^ (C=O) was measured at room temperature. Absorbance peak intensity values on the FTIR spectra were calculated using OMNIC 8.0 software (Spectra Tech, USA). The DC of the resin specimens was calculated using the following equation:

3 $${\rm{DC}}( \% )=1-[\frac{{(({{\rm{Abs}}}_{({\rm{C}}={\rm{C}})}{/\mathrm{Abs}}_{({\rm{C}}={\rm{O}})}))}_{{\rm{cured}}}}{{(({{\rm{Abs}}}_{({\rm{C}}={\rm{C}})}{/\mathrm{Abs}}_{({\rm{C}}={\rm{O}})}))}_{{\rm{Uncured}}}}]\times 100 \% $$

#### Flexural strength (FS) and flexural modulus (FM)

The FS and FM were tested according to ISO 4049: 2009 standards. PMMA resin specimens (2 × 2 × 25 mm) were prepared in a rectangular stainless steel mold. After curing, the specimens were removed from the mold and were placed in a water bath at 37 °C for 24 h. The three-point bending test was performed using a universal testing machine (AGS-10kNG, Shimadzu, Kyoto, Japan) at a cross-head speed of 0.05 mm/min at a controlled room temperature. The FS and FM were calculated by the following equations, respectively:

4 $${\rm{FS}}=3{\rm{FL}}/2{{\rm{wh}}}^{2}$$

5 $${\rm{FM}}={{\rm{kL}}}^{3}/4{{\rm{wh}}}^{3}$$

where F is the maximum load (N) exerted on the specimen, L is the distance (mm) between the supports (20 mm), w is the width of the specimen, h is the thickness of the specimen, and k is the slope of the line segment of the load/displacement graph. Mean FS and FM were calculated in megapascals (MPa). After testing, the microstructure of the fractured surfaces obtained from mechanical testing was observed with Fe-SEM.

#### Vickers microhardness

PMMA resin specimens (diameter: 6 mm, height: 3 mm) were prepared in a cylindrical stainless steel mold. After curing, the specimens were removed from the mold and were polished with No. 600, 1200, 2000, and 5000 abrasive paper, respectively. The specimens were then ultrasonically cleaned for 20 min before immersion in deionized water for 24 h at 37 °C. Vickers microhardness was measured by using a microhardness tester (HX-1000TM; Taiming, Shanghai, China). The indenter point was kept on the surface of specimens for 15 seconds with a 25-gram load. Three indentations were made on each specimen randomly. The experiments described above were repeated at least three times.

#### Statistical analysis

Statistical analyses were performed using SPSS 19.0 software. All data collected from this research was first checked for normal distribution (P = 0.05) with the Kolmogorov-Smirnov test. The antifungal ratio was analyzed with the Kruskal-Wallis H test and the Mann-Whitney U test at a correction significance level (α = 0.017). The remaining data sets were subjected to the Levene test for homogeneity of variance (P = 0.05), and one-way ANOVA and Tukey’s honestly significant difference tests (P = 0.05).

## Results

### Synthesis and characterization of AgBr/NPVP

**Figure 1:**
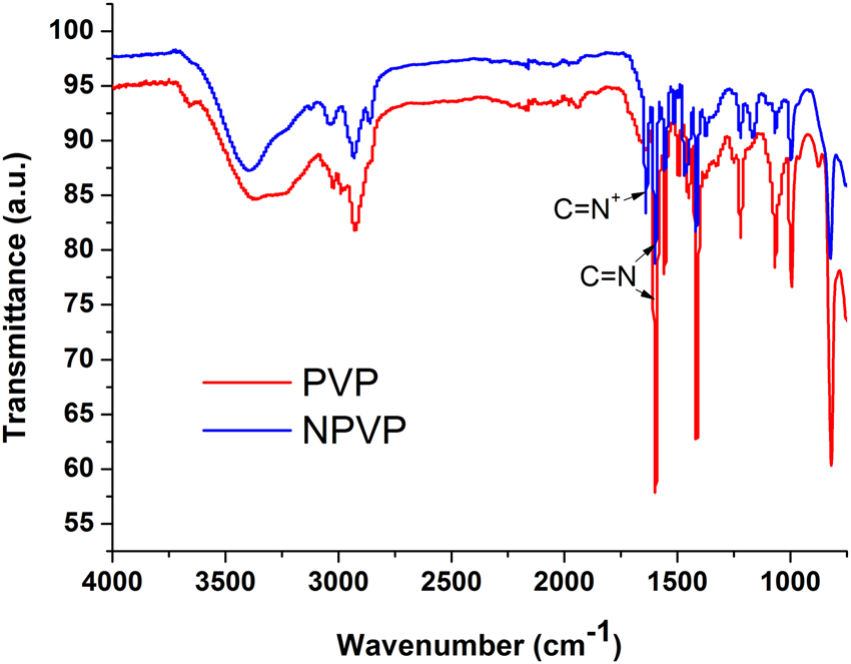
FTIR spectra of PVP and NPVP. The bands at 3400, 2900, 1467 and 1417 cm^−1^ are assigned to hydroxyl peaks, a methyl or methylene peak, and a pyridine ring C=C peak, respectively. The success of the N-alkylation was verified by the C=N^+^ stretching band at 1639 cm^−1^.

**Figure 2:**
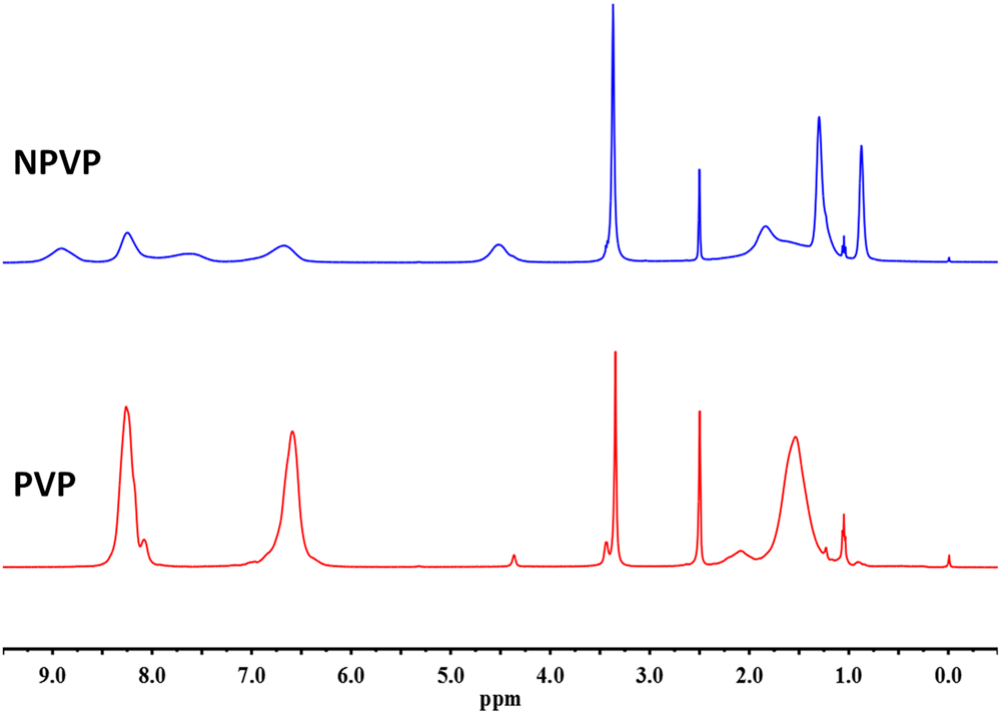
^1^HNMR spectra of PVP and NPVP. The peaks at 8.25 and 6.72 ppm were ascribed to the protons at the 2, 6 sites and 3, 5 sites of pyridine ring. After N-alkylation, they shifted to lower magnetic field of 7.68 and 8.91 ppm. Signal at 4.53 ppm was corresponding to the protons of the methylene groups bonded with N atom in the pyridine ring. The peaks from 1.31 to 1.86 ppm came from the aliphatic protons from the PVP backbone, the protons of methylene groups from alkyl bromides. The protons of methyl groups from alkyl bromides were observed in the high field of 0.88 ppm.

**Figure 3:**
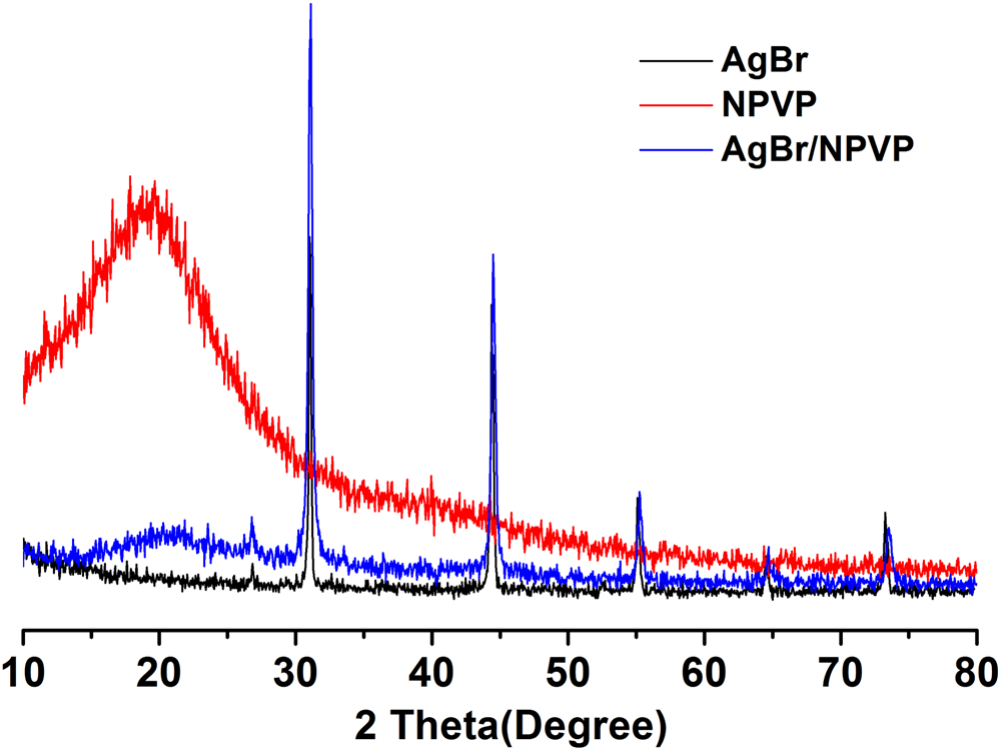
XRD patterns of AgBr, NPVP and AgBr/NPVP. The diffraction peaks located at 19.68° and 31.06°, 44.38°, 55.08°, 64.53°, 73.32° could be well indexed on the NPVP and AgBr crystal planes of (2 0 0), (2 2 0), (2 2 2), (4 0 0) and (4 2 0), respectively.

**Figure 4:**
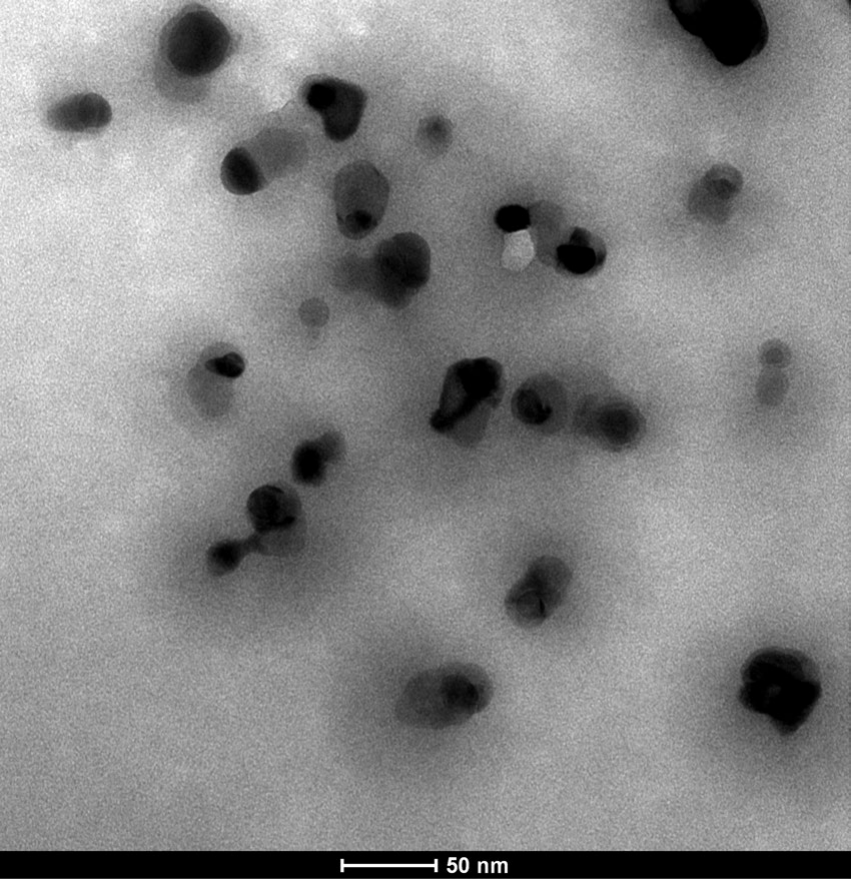
TEM images of AgBr/NPVP. The nanoparticle contains a single core and organic shell; that is, the spherical AgBr is embedded inside the cationic polymer NPVP, and the average diameter of AgBr/NPVP is approximately 30 nm.

FTIR (Fig. 1) was used to identify the surface organic groups of the PVP and NPVP. The bands at 3400, 2900, 1467 and 1417 cm^−1^ were assigned to hydroxyl peaks, a methyl or methylene peak, and a pyridine ring C=C peak, respectively. The success of the N-alkylation was verified by the C=N^+^ stretching band at 1639 cm^−1^. In order to further verify the intermediate products – PVP and NPVP composition, the samples were characterized by ^1^HNMR (Fig. 2). The peaks at 8.25 and 6.72 ppm were ascribed to the protons at the 2, 6 sites and 3, 5 sites of pyridine ring. After N-alkylation, they shifted to lower magnetic field of 7.68 and 8.91 ppm. Signal at 4.53 ppm was corresponding to the protons of the methylene groups bonded with N atom in the pyridine ring. The peaks from 1.31 to 1.86 ppm came from the aliphatic protons from the PVP backbone, the protons of methylene groups y6from alkyl bromides. The protons of methyl groups from alkyl bromides were observed in the high field of 0.88 ppm. The XRD diffraction patterns of AgBr/NPVP (Fig. 3) are consistent with the presence of AgBr and NPVP. The diffraction peaks located at 19.68° and 31.06°, 44.38°, 55.08°, 64.53°, 73.32° could be well indexed on the NPVP and AgBr crystal planes of (2 0 0), (2 2 0), (2 2 2), (4 0 0) and (4 2 0), respectively. The TEM images (Fig. 4) of AgBr/NPVP showed that the nanoparticle contains a single core and organic shell; that is, the spherical AgBr is embedded inside the cationic polymer NPVP, and the average diameter of AgBr/NPVP is approximately 30 nm.

### Determination of MIC and MFC

**Figure 5:**
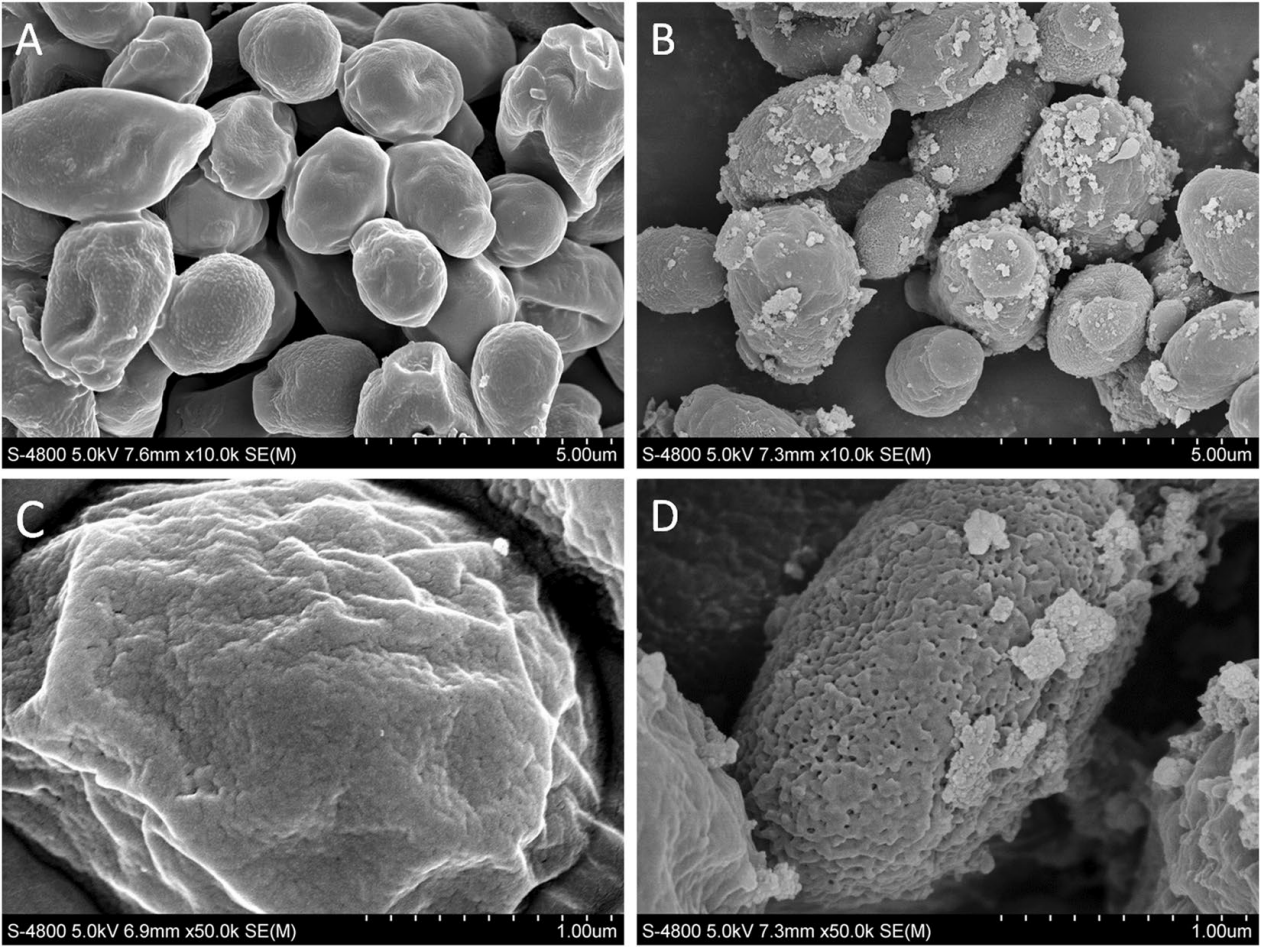
Fe-SEM images of C. albicans ATCC90028 morphology after incubation for 24 h. (**A**,**C**) Normal morphology of *C. albicans* ATCC90028. (**B**,**D**) Morphology of *C. albicans* ATCC90028 after incubation with AgBr/NPVP suspensions (1 × MFC) for 24 h.

The MIC and MFC values of AgBr/NPVP for *C. albicans* ATCC90028 were both 250 μg/ml. Normal cells displayed a regular morphology with a smooth, intact cell membrane (Fig. 5A,C). However, the integrity of the *C. albicans* cells was disturbed after contacting AgBr/NPVP (1 × MFC) for 24 h. The fungi lost their normal cell morphology, and there were pits and perforations on the surface of the cell membrane. Some cells were split into pieces (Fig. 5B,D).

### Antifungal ratio

**Figure 6:**
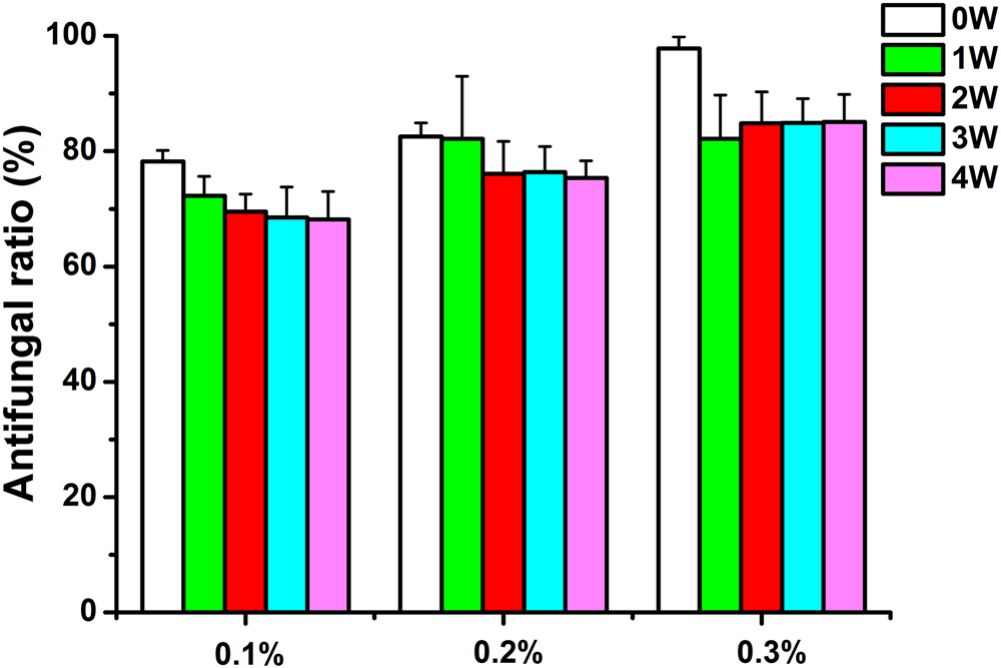
Histogram of the antifungal ratio of PMMA resin. Each value is the mean ± SD (n = 9). With the increase in AgBr/NPVP, the antifungal activity of unaged PMMA resin increased, and there were significant differences (P < 0.017) among the groups. The antifungal ratio of 1-week aging groups decreased compared with the corresponding unaged groups. Furthermore, the 0.3 wt% group decreased significantly. However, the antifungal ratio of each group showed a stable trend after 2 w of aging, and the 0.3 wt% group had the best antifungal effect, reaching more than 80%.

Figure 6 shows the antifungal ratio of the AgBr/NPVP-modified PMMA resin. The negative control and blank control groups had a large amount of fungal growth. The control group revealed no antifungal activity. The statistical results showed that when the dosage of AgBr/NPVP was 0.1, 0.2, and 0.3 wt%, the antifungal ratios of PMMA resin were (78.22 + 1.90)%, (82.58 + 2.35)%, and (97.82 + 2.05)%, respectively, before aging. With the increase of AgBr/NPVP, the antifungal activity of the PMMA resin increased, and there were significant differences (P < 0.017) among the groups. The antifungal ratio of the 1-week aging groups decreased relative to the corresponding unaged groups. Furthermore, the 0.3 wt% group decreased significantly. However, the antifungal ratio of each group showed a stable trend after 2 w of aging, and the 0.3 wt% group had the best antifungal effect, which reached more than 80%.

### CLSM analysis of fungal growth

**Figure 7:**
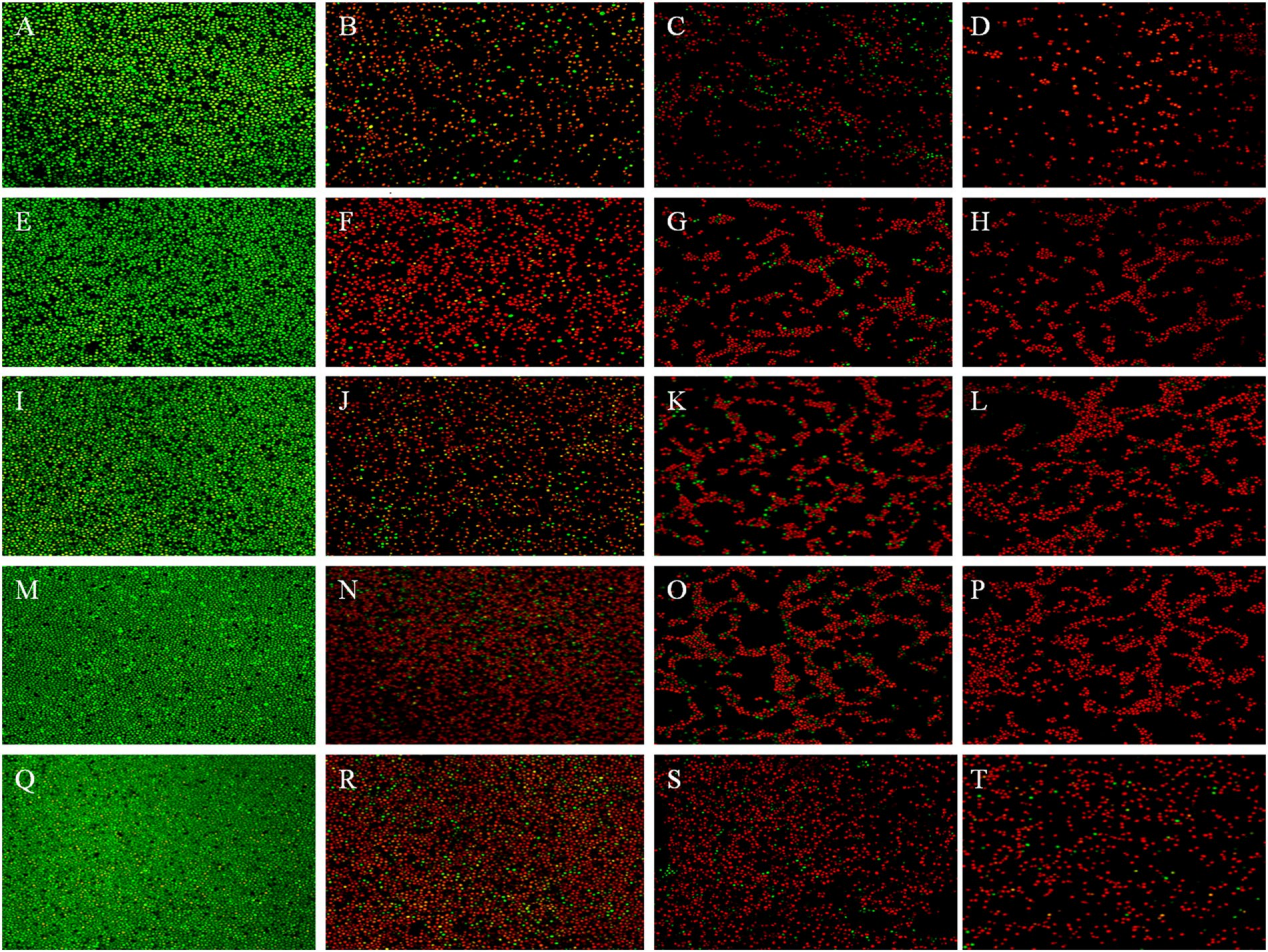
Live/dead staining of fungal biofilms on PMMA resin (×40). The transverse direction rows are the control (**A,E,I,M,Q**), 0.1 wt% (**B,F,J,N,R**), 0.2 wt% (**C,G,K,O,S**), and 0.3 wt% (**D,H,L,P,T**) groups, and the longitudinal direction columns are is the groups aged 0 w (**A,B,C,D**,), 1 w (**E,F,G,H**), 2 w (**I,J,K,L**), 3 w **(M,N,O,P**) and 4 w (**Q,R,S,T**). Live fungi were stained green, while dead fungi were stained red. Live/dead fungi that were close to or on top of each other produced yellow/orange colors. The control groups showed primarily live fungi. The AgBr/NPVP-containing PMMA resin showed substantial fungal activity.

Representative CLSM images of live/dead biofilms adhered to the PMMA resin specimens are shown in Fig. 7. The control groups (Fig. 7A,E,I,M,Q) were completely covered by dense and primarily live biofilms, especially in the 4-week aging groups (Fig. 7Q). Live fungi were stained green, and dead fungi were stained red. In some areas, the live and compromised fungi were closely associated; hence, the red color was mingled with green to yield yellow/orange colors. The experimental groups showed much more red/yellow/orange staining and much less fungi than the control groups, especially in the 0.3 wt% group of no aging (Fig. 7D). As the aging time prolonged, the attached fungi increased for each experimental group, while they were mainly stained red.

### Cytotoxicity Assay

**Figure 8:**
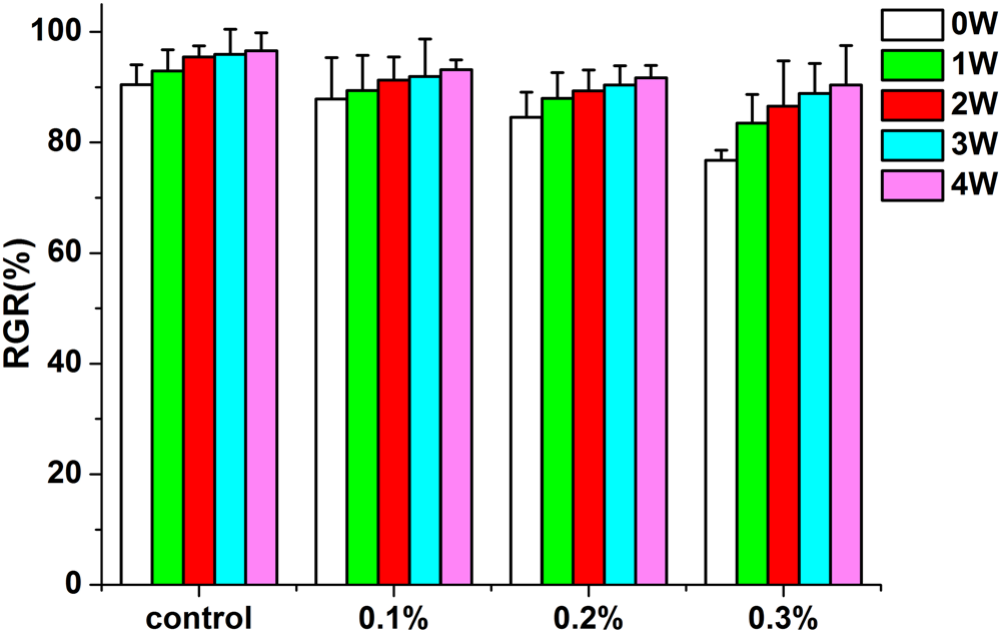
Cytotoxicity of PMMA resin with or without AgBr/NPVP. The values of the RGR ranged from (76.76 ± 1.84)% to (96.58 ± 3.23)%. The cytotoxicity of the PMMA resin decreased with the time and the decrease of AgBr/NPVP. When the dosage of AgBr/NPVP was 0.3 wt%, there were statistically difference (p < 0.05) between aging 3 and 4 w and the control group. There were no statistically differences between the groups with the rest dosages with the time (p > 0.05). There were statistically difference (p < 0.05) between 0.3 wt% and control group and 0.1 wt% group of before aging, also between 0.3 wt% and control group after aging 1 w. The rest aging groups had no statistical differences (p > 0.05) among different dosages.

Figure 8 illustrates the cell viability of PMMA resins containing different concentrations of AgBr/NPVP, which was determined as the RGR of cells compared with controls. The values of the RGR ranged from (76.76 ± 1.84)% to (96.58 ± 3.23)%. The cytotoxicity of the PMMA resin decreased with the time and the decrease of AgBr/NPVP. When the dosage of AgBr/NPVP was 0.3 wt%, there were statistically difference (p < 0.05) between aging 3 and 4 w and the control group. There were no statistically differences between the groups with the rest dosages with the time (p > 0.05). There were statistically difference (p < 0.05) between 0.3 wt% and control group and 0.1 wt% group of before aging, also between 0.3 wt% and control group after aging 1 w. The rest aging groups had no statistical differences (p > 0.05) among different dosages.

### Degree of Conversion

**Figure 9:**
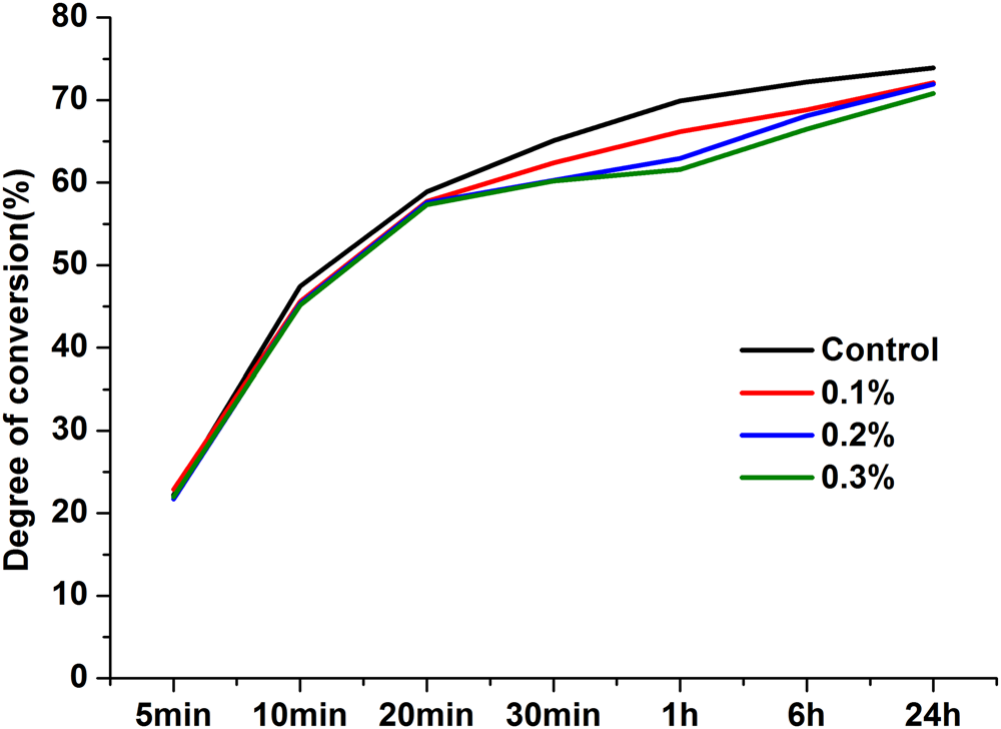
Degree of conversion of PMMA resin. The results are presented as the mean ± SD (n = 9). During the first 10 min curing period, DC increased dramatically, and after curing for 20 min, DC increased more slowly. At 24 h, the DC values of each group were all more than 70.0%, with no significant differences (P > 0.05) among the groups.

In Fig. 9, the DC was measured from 5 min to 24 h after the liquid - methyl methacrylate (MMA) was mixed with the PMMA powder. During the first 10 min curing period, DC increased dramatically, and DC increased less rapidly after 20 min of curing. At 1 h, the DC of the control and experimental subgroups ranged from (61.57 ± 1.96)% to (69.9 ± 1.25)%, and there was a significant difference (P < 0.05) between the 0.2 wt% and 0.3 wt% groups. At 24 h, the DC values of each group were all more than 70.0%, with no significant differences (P > 0.05).

### Mechanical properties

**Figure 10:**
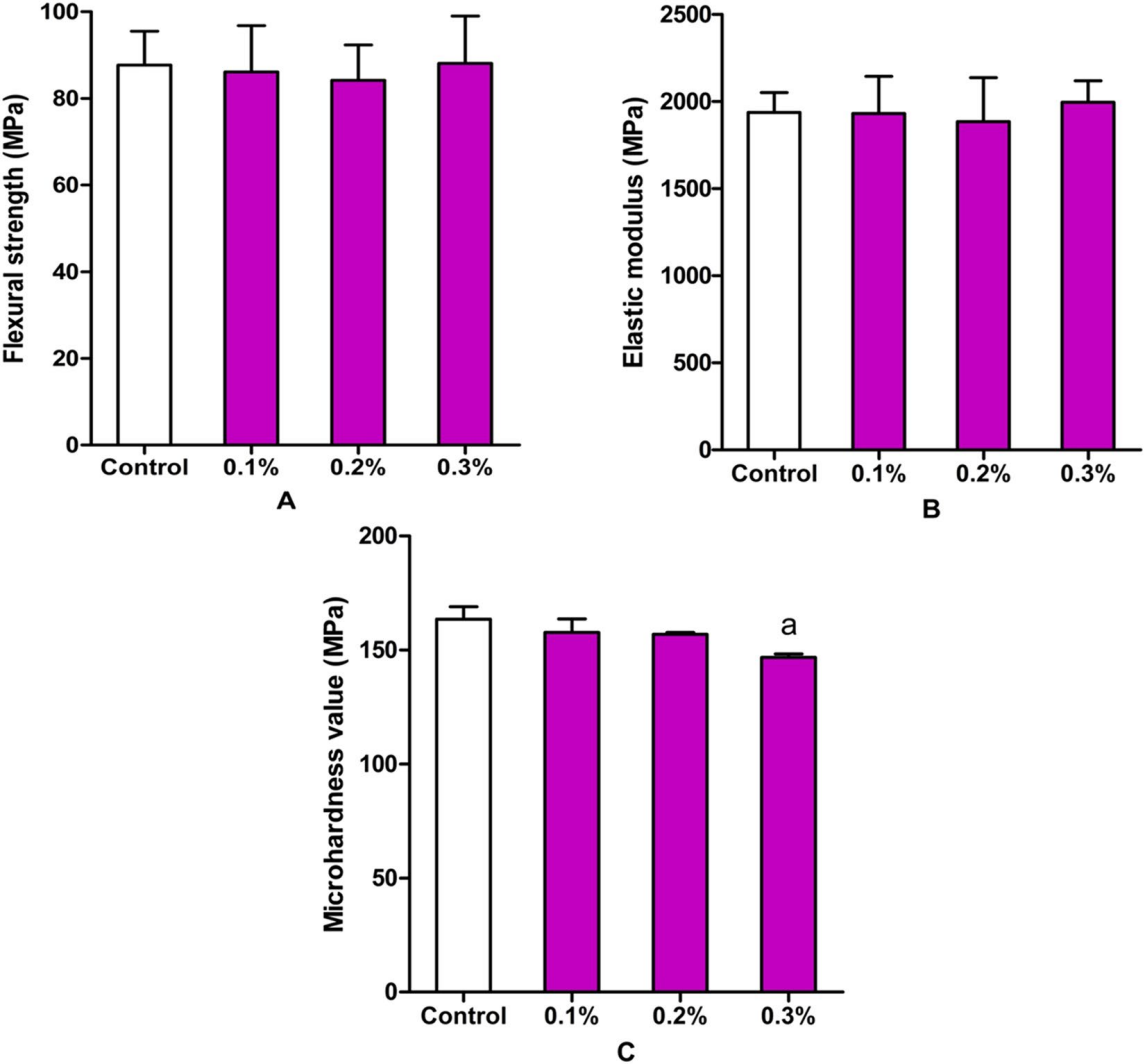
Mechanical properties of PMMA resin with or without AgBr/NPVP. (**A**) FS. (**B**) FM. (**C**) Vickers microhardness value. The data are presented as the mean ± SD (n = 9). “a” represents the significant difference (P < 0.05) with the other groups.

The FS, FM, and microhardness of the specimens in various subgroups after curing for 24 h are shown in Fig. 10. The incorporation of 0.1, 0.2, and 0.3 wt% AgBr/NPVP yielded no significant differences (P > 0.05) in the FS and FM of PMMA resin compared to the control group without AgBr/NPVP. However, when the concentration of the incorporated AgBr/NPVP was further increased, the microhardness of the materials showed a significantly reduced value at 0.3 wt% (P < 0.05).

### Surface morphology of fractured PMMA resin

**Figure 11:**
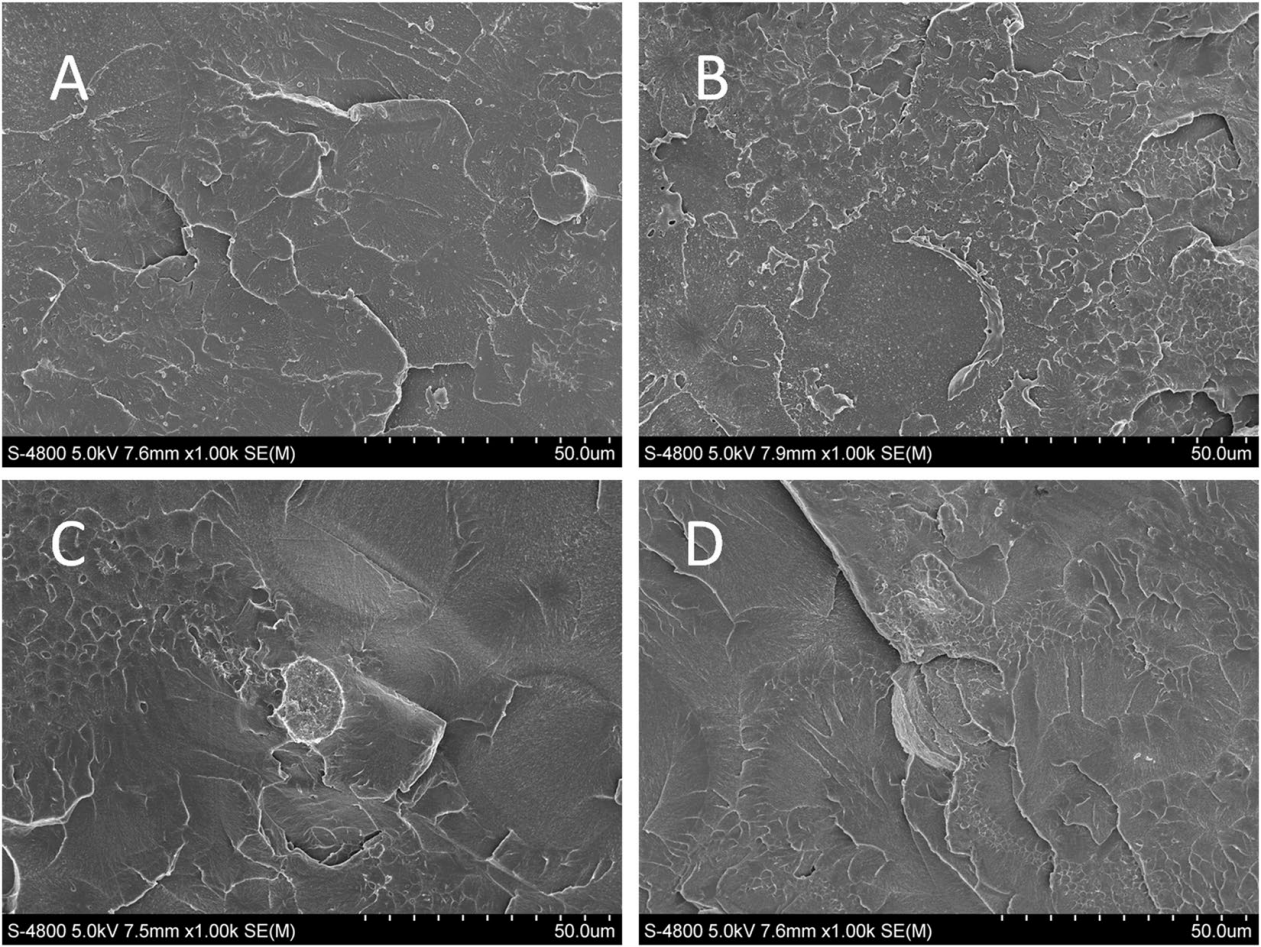
Fe-SEM images of fracture surfaces of (**A**) pure PMMA resin, and PMMA resin filled with different AgBr/NPVP loadings. (**B**) 0.1 wt%, (**C**) 0.2 wt%, (**D**) 0.3 wt%.

The FE-SEM images (Fig. 11) showed no obvious differences in the fractured surfaces of each group. There was a small hole on the fractured surface, as well as numerous rumples. The fractured surfaces of the control and samples containing 0.1 wt% to 0.3 wt% AgBr/NPVP exhibited brittle fractures with some sharp edge cracks and ductile cracks.

## Discussion

The essential factor in the formation of denture stomatitis is the adherence of *C. albicans* to the surfaces of denture base resins, followed by the formation of biofilms. Therefore, the key to prevent denture stomatitis is the control of *C. albicans* adhesion and biofilm formation. In this study, dual antimicrobial effects from silver ions and quaternary ammonium salts were obtained by incorporating the antimicrobial composite AgBr/NPVP into the denture base resin to prevent denture stomatitis. After microbial contact, silver ions adsorbed to the negatively charged surface of the microbes by Coulomb attraction, thus inhibiting the synthesis of cell wall peptidoglycan and undermining cell integrity. After passing through the cell walls, silver ions could interfere with the continuity of the cell membrane, increase its permeability, and affect the function of respiration and metabolism of microbes^30–34^. On the other hand, quaternary ammonium salt, which is the most widely used organic antimicrobial agent, could inhibit the free movement of microbes and their breathing by attracting the negatively charged cell membrane in so-called contact killing. Moreover, under the action of the electric field force, the distribution of the negative charge on cell walls and cell membranes was not homogeneous, which could cause the deformation and rupture of cells. In this case, cell contents such as water and protein would be lost, and cell death would occur via the phenomenon of “microbes dissolution”^35^. Changes in the cell morphology of *C. albicans* ATCC90028 after contacting the AgBr/NPVP (1 × MBC) suspension for 24 h under Fe-SEM observation were consistent with the mechanism mentioned above; the cells showed shrinkage, invagination, and content leakage, and some cells broke into pieces. Meanwhile, the limitation of quaternary ammonium salts was that the dead microbes and saliva protein could be adsorbed onto their surface, thus reducing the effectiveness of contact killing^36, 37^. However, AgBr/NPVP could release bioactive silver ions slowly, killing the microbes around the restoration without directly contacting them. Hence, AgBr/NPVP made up for the limitations of the use of quaternary ammonium salts alone. As shown in Figs 6 and 7, at the initial time, quaternary ammonium salts and silver ions exerted strong antifungal effects simultaneously, so the antifungal ratio and the ratio of dead / live fungi were relatively high. After the first week of aging, due to the relatively rapid release of silver ions, the decline in the antifungal ratio was relatively obvious. In the second week of aging and later, the release of silver ions became stable, and NPVP incorporated into PMMA resins also had stable antifungal activity. Therefore, the antifungal effects of AgBr/NPVP-modified PMMA resins tended to be stable. Consequently, modified PMMA resins not only had a contact inhibition effect but also showed a far-reaching antimicrobial effect^28^.

Cytotoxicity was rated in accordance with ISO-standard 10993–5 as non-cytotoxic (cell viability higher than 75%), slightly cytotoxic (cell viability ranging from 50% to 75%), moderately cytotoxic (cell viability ranging from 25% to 50%), and severely cytotoxic (cell viability lower than 25%). The results showed that AgBr/NPVP-modified PMMA resins were non-cytotoxic towards HDPCs according to the standard. It is well accepted that polymerization of dental resinous materials is never complete^38, 39^ and the unpolymerized monomers can be released over time. The unpolymerized monomers can be the primary reason for the cytotoxicity of dental resinous materials^40^. In case of PMMA resin, its cytotoxicity can be attributed to the release of unpolymerized MMA monomer. Similar to other methacylate monomers, MMA can disturb the intracellular redox balance and thus inducing negative influences on the function and viability of cells^40–44^. As shown in Fig. 8, the toxicity of control group decreased gradually with the time and the release of monomer, and the toxicity was mostly derived from the MMA monomer. However, the toxicity of AgBr/NPVP-modified PMMA groups were mostly derived from the release of not only the MMA monomer but also silver ions, so the toxicity of AgBr/NPVP-modified PMMA groups was slightly larger than that of the control group with the same aging time, and the toxicity increased with the increase of the dosage of AgBr/NPVP.

The conversion of a monomer into a polymer during the polymerization reaction can be reflected by the DC. Although DC is not the only factor that determines the mechanical properties of dental polymers, lower DC is usually associated with poorer mechanical properties. In our study, Fig. 9 showed that the polymerization of the PMMA resin was a time-dependent reaction. Within the first ten minutes after mixing, the specimens polymerized at a relatively fast rate, and the DC reached a high level after this period. The DC for the control group reached (47.43 ± 0.80)% after 10 min. In addition, the incorporation of AgBr/NPVP reduced the DC in a dose-dependent manner, with the subgroups containing 0.1, 0.2, and 0.3 wt% AgBr/NPVP presenting DC values of (45.60 ± 0.62)%, (45.33 ± 0.91)%, and (45.13 ± 1.88)%, respectively, and no significant difference was noted among groups (P > 0.05). However, in the second 10 min and later, the ratio of polymerization decreased slowly. In the following period, from 20 min to 24 h, the polymerization process of all subgroups maintained a stable rate of slow polymerization. At 24 h, the DC values for all subgroups were above 70%, with (73.90 ± 1.11)%, (72.12 ± 0.95)%, (71.93 ± 0.85)% and (70.80 ± 2.40)%, and there were no significant differences among the groups. These results are supported by previous studies, which found that the greatest increase in DC occurs within the first 10 min after mixing the dental resins^26, 27^. However, the reduction of DC related to AgBr/NPVP-incorporating PMMA resin might be attributed to the creation of a heterogeneous system with a reduced local concentration of MMA by unpolymerized AgBr/NPVP.

Dentures are prone to fracture during use, and the FS has slight changes in sensitivity to the sub-structure of the material^45^, so the FS was used as one of the indices of the evaluation of the mechanical properties of modified PMMA resin. In this study, the FS results showed that there were no significant effects for 3 dosages of the AgBr/NPVP on the FS of PMMA resin, and the surface morphology of the fractured PMMA resin also supported the above conclusion. The FM measures the rigidity of the material, and the results of this study showed that the 3 samples with different added dosages of AgBr/NPVP had no significant effect on the FM of the PMMA resin. On the other hand, hardness is the mechanical property of the material that determines its resistance to force. The Vickers microhardness of PMMA resin without adding AgBr/NPVP was (163.54 ± 5.49) MPa. With the increased addition of AgBr/NPVP, the microhardness decreased gradually, and when adding 0.3 wt%, the microhardness was reduced to (146.80 ± 1.46) MPa, which was significantly different from that of the other groups (P < 0.05).

## Conclusions

In this study, AgBr/NPVP was synthesized and used in PMMA resin as a reactant antibacterial agent. The results indicate that in comparison with the control groups, AgBr/NPVP-modified PMMA resins have antifungal activities before or after 1, 2, 3, or 4 w of aging. The antifungal activity of the resins increased with increasing AgBr/NPVP content. Even at low concentrations of AgBr/NPVP, as low as 0.1 wt% or 0.2 wt%, the modified PMMA resins showed sufficient antifungal activity and reduced fungal growth in comparison to resin samples without AgBr/NPVP, but they had low toxicity toward HDPCs and did not affect mechanical properties. However, the microhardness of specimens with 0.3 wt% AgBr/NPVP was significantly lower. Therefore, the AgBr/NPVP concentration selected for some applications may need to balance the antifungal effects with mechanical properties. At the same time, further research should be focused on the study of the biocompatibility of AgBr/NPVP-modified PMMA resins.

## References

[1] Al-Dwairi ZN (2008). Prevalence and risk factors associated with denture-related stomatitis in healthy subjects attending a dental teaching hospital in North Jordan. Journal of the Irish Dental Association.

[2] Budtz-Jlrgensen E, Mojon P, Banon-Clement JM, Baehni P (1996). Oral candidosis in long-term hospital care: comparison of edentulous and dentate subjects. Oral diseases.

[3] Gendreau L, Loewy ZG (2011). Epidemiology and etiology of denture stomatitis. Journal of prosthodontics: official journal of the American College of Prosthodontists.

[4] Budtz-Jorgensen E, Mojon P, Rentsch A, Deslauriers N (2000). Effects of an oral health program on the occurrence of oral candidosis in a long-term care facility. Community dentistry and oral epidemiology.

[5] Jeganathan S, Lin CC (1992). Denture stomatitis–a review of the aetiology, diagnosis and management. Australian dental journal.

[6] Batista JM, Birman EG, Cury AE (1999). Suscetibilidade a antifúngicos de cepas de Candida albicans isoladas de pacientes com estomatite protética. Rev. Odontol. Univ. São Paulo.

[7] Skupien JA, Valentini F, Boscato N, Pereira-Cenci T (2013). Prevention and treatment of Candida colonization on denture liners: a systematic review. The Journal of prosthetic dentistry.

[8] Falah-Tafti A, Jafari AA, Lotfi-Kamran MH, Fallahzadeh H, Hayan RS (2010). A comparison of the efficacy of nystatin and fluconazole incorporated into tissue conditioner on the *in vitro* attachment and colonization of Candida albicans. Dental research journal.

[9] Monteiro DR, Gorup LF, Takamiya AS, de Camargo ER, Barbosa DB (2012). Silver distribution and release from an antimicrobial denture base resin containing silver colloidal nanoparticles. Journal of Prosthodontics.

[10] Gosheger G (2004). Silver-coated megaendoprostheses in a rabbit model–an analysis of the infection rate and toxicological side effects. Biomaterials.

[11] Percival SL, Bowler PG, Russell D (2005). Bacterial resistance to silver in wound care. The Journal of hospital infection.

[12] Chaloupka K, Malam Y, Seifalian AM (2010). Nanosilver as a new generation of nanoproduct in biomedical applications. Trends in biotechnology.

[13] Pal S, Tak YK, Song JM (2007). Does the antibacterial activity of silver nanoparticles depend on the shape of the nanoparticle? A study of the Gram-negative bacterium Escherichia coli. Applied and environmental microbiology.

[14] Seth D (2011). Nature-inspired novel drug design paradigm using nanosilver: efficacy on multi-drug-resistant clinical isolates of tuberculosis. Current microbiology.

[15] Li Z, Sun J, Lan J, Qi Q (2016). Effect of a denture base acrylic resin containing silver nanoparticles on Candida albicans adhesion and biofilm formation. Gerodontology.

[16] Wady AF (2012). Evaluation of Candida albicans adhesion and biofilm formation on a denture base acrylic resin containing silver nanoparticles. Journal of applied microbiology.

[17] Durner J, Stojanovic M, Urcan E, Hickel R, Reichl FX (2011). Influence of silver nano-particles on monomer elution from light-cured composites. Dental materials: official publication of the Academy of Dental Materials.

[18] Cheng L (2012). Antibacterial amorphous calcium phosphate nanocomposites with a quaternary ammonium dimethacrylate and silver nanoparticles. Dental materials: official publication of the Academy of Dental Materials.

[19] Cheng L (2012). Effect of amorphous calcium phosphate and silver nanocomposites on dental plaque microcosm biofilms. Journal of biomedical materials research. Part B, Applied biomaterials.

[20] Buffet-Bataillon S, Tattevin P, Bonnaure-Mallet M, Jolivet-Gougeon A (2012). Emergence of resistance to antibacterial agents: the role of quaternary ammonium compounds–a critical review. International journal of antimicrobial agents.

[21] Gilbert P, Moore LE (2005). Cationic antiseptics: diversity of action under a common epithet. Journal of applied microbiology.

[22] Pesci-Bardon C, Fosse T, Madinier I, Serre D (2004). *In vitro* new dialysis protocol to assay the antiseptic properties of a quaternary ammonium compound polymerized with denture acrylic resin. Letters in applied microbiology.

[23] Pesci‐Bardon C, Fosse T, Serre D, Madinier I (2006). *In vitro* antiseptic properties of an ammonium compound combined with denture base acrylic resin. Gerodontology.

[24] Caillier L (2009). Synthesis and antimicrobial properties of polymerizable quaternary ammoniums. European journal of medicinal chemistry.

[25] McDonnell G, Russell AD (1999). Antiseptics and disinfectants: activity, action, and resistance. Clinical microbiology reviews.

[26] Balkenhol M, Ferger P, Mautner MC, Wostmann B (2007). Provisional crown and fixed partial denture materials: mechanical properties and degree of conversion. Dental materials: official publication of the Academy of Dental Materials.

[27] Ferracane JL (1985). Correlation between hardness and degree of conversion during the setting reaction of unfilled dental restorative resins. Dental Materials.

[28] Sambhy V, MacBride MM, Peterson BR, Sen A (2006). Silver bromide nanoparticle/polymer composites: dual action tunable antimicrobial materials. Journal of the American Chemical Society.

[29] Makvandi P, Ghaemy M, Ghadiri AA, Mohseni M (2015). Photocurable, Antimicrobial Quaternary Ammonium-modified Nanosilica. Journal of dental research.

[30] Galdiero S (2011). Silver nanoparticles as potential antiviral agents. Molecules.

[31] Morones JR (2005). The bactericidal effect of silver nanoparticles. Nanotechnology.

[32] Feng Q (2000). A mechanistic study of the antibacterial effect of silver ions on Escherichia coli and Staphylococcus aureus. Journal of biomedical materials research.

[33] Jain J (2009). Silver nanoparticles in therapeutics: development of an antimicrobial gel formulation for topical use. Molecular Pharmaceutics.

[34] Kim JS (2007). Antimicrobial effects of silver nanoparticles. Nanomedicine: nanotechnology, biology, and medicine.

[35] Namba N (2009). Antibacterial effect of bactericide immobilized in resin matrix. Dental Materials.

[36] Imazato S (2003). Antibacterial properties of resin composites and dentin bonding systems. Dental Materials.

[37] Beyth N, Yudovin-Farber I, Bahir R, Domb AJ, Weiss EI (2006). Antibacterial activity of dental composites containing quaternary ammonium polyethylenimine nanoparticles against Streptococcus mutans. Biomaterials.

[38] Kopperud HM, Kleven IS, Wellendorf H (2011). Identification and quantification of leachable substances from polymer-based orthodontic base-plate materials. European journal of orthodontics.

[39] Miletic V, Santini A, Trkulja I (2009). Quantification of monomer elution and carbon-carbon double bonds in dental adhesive systems using HPLC and micro-Raman spectroscopy. Journal of dentistry.

[40] Att W, Yamada M, Kojima N, Ogawa T (2009). N-Acetyl cysteine prevents suppression of oral fibroblast function on poly(methylmethacrylate) resin. Acta biomaterialia.

[41] Ciapetti G (2002). *In vitro* testing of the potential for orthopedic bone cements to cause apoptosis of osteoblast-like cells. Biomaterials.

[42] Huang FM, Chang YC (2005). Prevention of the epoxy resin-based root canal sealers-induced cyclooxygenase-2 expression and cytotoxicity of human osteoblastic cells by various antioxidants. Biomaterials.

[43] Ratanasathien S, Wataha JC, Hanks CT, Dennison JB (1995). Cytotoxic interactive effects of dentin bonding components on mouse fibroblasts. J Dent Res.

[44] Vale FM (1997). Acrylic bone cement induces the production of free radicals by cultured human fibroblasts. Biomaterials.

[45] Yap A, Teoh S (2003). Comparison of flexural properties of composite restoratives using the ISO and mini‐flexural tests. Journal of oral rehabilitation.

